# Perinatal and pediatric outcomes associated with the use of fertility treatment: a population-based retrospective cohort study in Ontario, Canada

**DOI:** 10.1186/s12884-023-05446-3

**Published:** 2023-02-20

**Authors:** Sheryll Dimanlig-Cruz, Daniel J. Corsi, Andrea Lanes, Lynn Meng, Qun Miao, Mark Walker, Deshayne B. Fell

**Affiliations:** 1 Children’s Hospital of Eastern Ontario (CHEO) Research Institute, Ottawa, ON Canada; 2 Better Outcomes Registry & Network (BORN) Ontario, Ottawa, ON Canada; 3 School of Epidemiology and Public Health, University of Ottawa, Ottawa, ON Canada; 4 OMNI Research Group, Ottawa Hospital Research Institute, Ottawa, ON Canada; 5 Department of Obstetrics and Gynecology, University of Ottawa Faculty of Medicine, Ottawa, ON Canada

**Keywords:** Assisted reproductive technology, Ovulation induction, Intra-uterine insemination, Perinatal health, Pediatric health

## Abstract

**Background:**

Around 2% of births in Ontario, Canada involve the use of assisted reproductive technology (ART), and it is rising due to the implementation of a publicly funded ART program in 2016. To better understand the impact of fertility treatments, we assessed perinatal and pediatric health outcomes associated with ART, hormonal treatments, and artificial insemination compared with spontaneously conceived births.

**Methods:**

This population-based retrospective cohort study was conducted using provincial birth registry data linked with fertility registry and health administrative databases in Ontario, Canada. Live births and stillbirths from January 2013 to July 2016 were included and followed to age one. The risks of adverse pregnancy, birth and infant health outcomes were assessed by conception method (spontaneous conception, ART – in vitro fertilization and non-ART – ovulation induction, intra-uterine or vaginal insemination) using risk ratios and incidence rate ratios with 95% confidence intervals (CI). Propensity score weighting using a generalized boosted model was applied to adjust for confounding.

**Result(s):**

Of 177,901 births with a median gestation age of 39 weeks (IQR 38.0–40.0), 3,457 (1.9%) were conceived via ART, and 3,511 (2.0%) via non-ART treatments. There were increased risks (adjusted risk ratio [95% CI]) of cesarean delivery (ART: 1.44 [1.42–1.47]; non-ART: 1.09 [1.07–1.11]), preterm birth (ART: 2.06 [1.98–2.14]; non-ART: 1.85 [1.79–1.91]), very preterm birth (ART: 2.99 [2.75–3.25]; non-ART: 1.89 [1.67–2.13]), 5-min Apgar < 7 (ART: 1.28 [1.16–1.42]; non-ART: 1.62 [1.45–1.81]), and composite neonatal adverse outcome indicator (ART: 1.61 [1.55–1.68]; non-ART: 1.29 [1.25–1.34]). Infants born after fertility treatments had increased risk of admission to neonatal intensive care unit (ART: 1.98 [1.84–2.13]; non-ART: 1.59 [1.51–1.67]) and prolonged birth admission (≥ 3 days) (ART: 1.60 [1.54–1.65]; non-ART: 1.42 [1.39–1.45]). The rate of emergency and in-hospital health services use within the first year was significantly increased for both exposure groups and remained elevated when limiting analyses to term singletons.

**Conclusion(s):**

Fertility treatments were associated with increased risks of adverse outcomes; however, the overall magnitude of risks was lower for infants conceived via non-ART treatments.

**Supplementary Information:**

The online version contains supplementary material available at 10.1186/s12884-023-05446-3.

## Introduction

Infertility affects about 186 million individuals globally [1] and consequently, demand for fertility treatments is high [2]. Assisted reproductive technology (ART) encompass procedures involving in vitro handling of human oocytes and sperm or embryo [3] and in vitro fertilization (IVF) is the most common ART procedure, with an estimated 2.5 million treatment cycles performed globally each year, resulting in more than 500,000 babies [2, 4].

Studies have reported increased risks of adverse pregnancy and birth outcomes following ART, compared with spontaneously conceived pregnancies: multiple birth, preterm birth, small for gestational age (SGA), low birthweight, stillbirth, induction of labor, and cesarean delivery [5–10]. ART has also been associated with low Apgar scores, admission to neonatal intensive care, and infant mortality [11–13]. However, most existing ART studies have classified infants born after non-ART fertility treatments (hormonal treatments and insemination) as spontaneously conceived infants and have focused on risks of ART alone; studies evaluating pregnancy and infant health outcomes following non-ART fertility treatments are limited.

Studies examining longer-term health of children conceived via ART suggest that they are generally healthy and develop normally. Based on three systematic reviews, no difference was apparent in the overall development of ART and spontaneously conceived children [14–16]. A national cohort study of ART children in the UK reported early childhood growth patterns similar to those of spontaneously conceived children [17]. However, some evidence has demonstrated possible associations of conception with ART with congenital malformations, genetic disorders, greater use of hospital services, and longer hospitalizations [18–21]. Long-term follow-up studies have observed increased risk of cardiovascular diseases, diabetes and certain type of cancers, although the results may have been confounded by genetic characteristics and lifestyle factors [22–24].

Between 2013 and 2019, conceptions through ART alone accounted for 21,003 births in Ontario, Canada [25] and the use of ART has increased substantially due to the introduction of a publicly funded ART program (Ontario Fertility Program) in 2016 [25, 26]. This provides a significant opportunity to better understand the impact of fertility treatments on maternal and child health.

In this study, we assessed pregnancy, birth, and infant health outcomes up to age one in Ontario, Canada. We compared pregnancies conceived via ART and non-ART treatments (ovulation induction, intra-uterine insemination, vaginal insemination) with spontaneous conceptions using Ontario’s birth registry, linked with the ART registry and other provincial health administrative databases.

## Methods

### Study design and population

This was a population-based retrospective study. The study cohort comprised all live and stillbirths (singletons and multiple births) in Ontario, Canada conceived between January 11, 2013 and July 7, 2016. For ART pregnancies, we determined the date of conception using embryo transfer date. For pregnancies conceived via non-ART fertility treatments and for spontaneously conceived pregnancies, we added 14 days to the date of the last menstrual period in the Better Outcomes Registry & Network (BORN) Information System (BIS). Additional file 1 shows the timeline of the first and last eligible conceptions, with corresponding births and the one-year follow-up window.

The study included women who delivered live or stillborn infants at ≥ 20 weeks’ gestation or with a birthweight of ≥ 500 g. We excluded pregnant people younger than age 18; heterotopic, ectopic and molar pregnancies; elective terminations; and pregnancies that used donor oocytes or gestational surrogates. For singleton deliveries, records with missing birthweight and unclassified sex were removed (< 0.01%) and an algorithm [27, 28] was applied to identify newborns with implausible birthweight/gestational age combinations; these records were excluded.

### Data sources

We analyzed maternal and newborn health information from the BORN Ontario birth registry, which contains data on clinical encounters in more than 250 hospitals, fertility clinics, birth centres, midwifery practice groups, primary-care organizations, and other health care providers [29]. The BIS captures data from conception through birth and into the newborn period, including maternal demographics and health behaviours, obstetric history, and clinical information about pregnancy, labor and delivery, and neonatal outcomes [29]. Based on a data quality re-abstraction study, 22 out of 29 of the audited variables showed excellent agreement with patient medical charts (> 90%); the remaining 7 had fair-to-moderate agreement [30]. An external audit by Public Health Ontario confirmed the high accuracy of BIS data [31].

Housed in BORN Ontario, the Canadian Assisted Reproductive Technologies Register (CARTR) Plus contains information on IVF treatment cycles from 97% of fertility clinics across Canada [32]. CARTR Plus captures patient demographics and obstetric history, clinical information about treatment cycles (reason, number of follicles, endometrial thickness, and embryo transfer date), and outcome of treatment cycles. Ontario treatment cycle records are linked with BIS birth records, thereby providing information on pregnancy and birth outcomes for live and stillbirths. A validation study of CARTR Plus found that agreement of assessed variables with medical chart re-abstraction ranged from 62.1% to 99.9%; 68% of assessed variables had more than 90% agreement [33].

We used the Canadian version of International Classification of Diseases, 10th Revision (ICD-10-CA) codes for medical diagnosis and the Canadian Classification of Health Interventions codes for clinical procedures from the Canadian Institute for Health Information’s (CIHI) Discharge Abstract Database (DAD) and the National Ambulatory Care Reporting System (NACRS) to ascertain pediatric outcomes during hospital admissions and emergency department visits up to age one. The DAD contains administrative, clinical, and demographic information from hospital separation abstracts. The NACRS contains data from hospital-based and community-based ambulatory care. All Ontario acute care facilities submit inpatient and ambulatory care visit data to CIHI and a file of Ontario hospital abstracts for obstetric deliveries and infant health services use up to age one is transferred to BORN Ontario each year.

### Exposure assessment

Pregnant individuals were categorized into three mutually exclusive groups based on conception method: (1) spontaneous conception; (2) ART—IVF (with or without intracytoplasmic sperm injection, with autologous oocytes either from fresh IVF or frozen embryo transfer cycles); and (3) non-ART – fertility treatments including ovulation induction, intra-uterine or vaginal insemination. Differentiation of the latter two groups was based on definitions set by the International Committee for Monitoring Assisted Reproductive Technologies [3]. We used CARTR Plus to identify pregnancies conceived using ART (Group 2), and BIS to classify the remaining records as spontaneous conception (Group 1) or conception via non-ART (Group 3).

### Outcome assessment

#### Pregnancy and birth outcomes

Pregnancy and birth outcomes were obtained from BIS data, except for the composite neonatal adverse outcome indicator (NAOI), which was ascertained from DAD diagnoses and procedure codes (see Additional file 2). Binary variables (yes/no) were created for: stillbirth, multiple birth, cesarean delivery, preterm birth (< 37 weeks), very preterm birth (< 32 weeks), SGA birth (< 10^th^ percentile for sex- and gestational age-specific birthweight), 5-min APGAR score < 7, and an adaptation of the composite NAOI, which measures severe neonatal morbidity within the first 28 days of life [34, 35]. SGA infants (only singleton live births) were identified based on a Canadian reference standard [27].

#### Infant health services use and health outcomes

Using data from DAD and NACRS, we assessed outcomes among infants from birth to age one: (1) non-specific and disease-specific health services use, and (2) health outcomes. Non-specific health services use included admission to neonatal intensive care unit (NICU) for more than 24 h (yes/no), and 3 or more days of hospital stay for the birth admission (yes/no). Additional non-specific outcomes were rates of all-cause hospitalization and emergency department visits during the first year. An episode of care was the unit of analysis and defined as all contiguous hospitalizations; therefore, repeat emergency department visits within 24-h of a previous visit for the same diagnosis, transfers from ambulatory to inpatient care, and inter-hospital transfers for admission were considered as one episode of care. Measures of specific disease-related health services use were hospitalization rates for upper and lower respiratory tract infections, gastrointestinal infections, otitis media, and a composite of these infections (see Additional file 3). Infant health outcomes were pediatric chronic disease (composite indicator for pediatric complex chronic conditions [PCCC]) and infant death (discharge disposition on hospitalization abstracts). PCCC identifies children with life-limiting illnesses that are expected to last at least 12 months and require specialty pediatric care [36] and we classified infants as having two or more complex chronic conditions versus none or one (yes/no) (see Additional file 4).

### Covariates

Several baseline maternal characteristics were potential confounders: age at delivery (years), neighbourhood household income and education level (by quintile); body mass index (BMI) category (≥ 30 kg/m^2^ as obese, < 30 kg/m^2^ as non-obese), gravidity; parity; pre-pregnancy health conditions (yes/no; asthma, diabetes, chronic hypertension), health complications during pregnancy (yes/no; gestational diabetes, hypertensive disorders), and adverse health behaviours during pregnancy (yes/no; smoking, alcohol consumption, illicit drug use). Neighbourhood household income and education level were derived from Statistics Canada’s Census of Population.

### Statistical analysis

#### Descriptive analyses

We assessed the frequency distribution of baseline maternal characteristics across conception groups and compared women who had conceived via ART or non-ART fertility treatments with their spontaneous conception counterparts, based on standardized differences. An absolute value of < 0.10 indicated a well-balanced baseline covariate (ART vs. spontaneous conception, non-ART vs. spontaneous conception).

#### Multiple imputation

The prevalence of missing data across covariates ranged between 0% and 14.1%. Assuming that data was missing at random, we used the fully conditional specification (FCS) approach [37] within the PROC MI procedure in SAS (Version 9.4, SAS Institute Inc., Cary, NC) to impute missing values. We created 10 imputed datasets with maternal covariates in our imputation model.

#### Propensity score models

We used the generalized boosted model (GBM) to estimate propensity scores and their associated weights [38]. Compared with logistic regression models, GBM results in a better balance of covariates and treatment effect estimators, with smaller mean-squared error [39, 40].

We used the Toolkit for Weighting and Analysis of Non-equivalent Groups (TWANG) R package to estimate propensity score weights, incorporating the same covariates in multiple imputation. We set a maximum of 7,000 iterations (regression trees) and used the sum of effect sizes across all covariates for optimization when fitting our propensity score models. We also used the average treatment effect (ATE) to estimate treatment effects in the entire study population. The ATE weights were considered as propensity score weights, which were integrated into all regression models to generate adjusted parameter estimates. Box plots were used to compare the distributions and assess overlap of ATE weights between reference and exposure groups. ATE weights were capped (“winsorized”) at 0.01^st^ and 99.99^th^ percentile to deal with extreme values and prevent variance inflation.

#### Regression analyses

Associations between fertility treatments and stillbirth, multiple birth, cesarean delivery, and preterm birth were examined among all live and stillbirths (Cohort 1); the remaining outcomes were assessed among live births only (Cohort 2). We computed cumulative incidences (binary outcomes) and incidence rates per 1,000 person-days of follow-up (count outcomes). For the latter, follow-up of each infant began on the date of birth and continued until either death or age one. We used log-binomial regression models for binary outcomes and Poisson generalized linear models for count outcomes (analyze as rates) to generate unadjusted and adjusted risk ratios (RR; aRR) and unadjusted and adjusted incidence rate ratios (IRR; aIRR), respectively, with 95% confidence intervals (CI). The Poisson models of rates included the total count of outcome events with an offset of the log of person-days and scaled by deviance to generate rates and address overdispersion of data. Parameter estimates were computed separately for each imputed dataset. All results were then pooled into a single estimate using PROC MIANALYZE.

#### Sensitivity analyses

To determine if outcomes were influenced by multiple births in this study cohort, we restricted our analyses for pregnancy and birth outcomes to singletons. Analyses for infant health outcomes were further limited to full-term singletons to determine if associations between fertility treatments and infant health were influenced by plurality and prematurity. We only assessed outcomes that were statistically significant from the primary analysis.

We used SAS 9.4 to perform statistical analyses and R (version 4.0) to run propensity score models.

This study received ethical approval from the Children’s Hospital of Eastern Ontario Research Ethics Board (20/12PE) and was also approved by the BORN Ontario Research Review Committee. This study involved secondary use of databases housed at BORN Ontario; therefore, individual patient consent was not required. As a Prescribed Registry under the Personal Health Information Protection Act (PHIPA), BORN Ontario has the authority to collect, use, and disclose personal health information without patient consent for the purpose of facilitating and improving the provision of health care. Data management and analysis for this study was conducted within the secure network environment at BORN Ontario and followed all required privacy and security policies as stipulated by PHIPA legislation and BORN Ontario. All methods were performed in accordance with the local relevant guidelines and regulations, as well as in accordance with the Declaration of Helsinki. This study followed the REporting of studies Conducted using Observational Routinely-collected Data (RECORD) guidelines for reporting, as outlined in https://www.record-statement.org/checklist.php (see Additional file 5) [41].

## Results

**Fig. 1:**
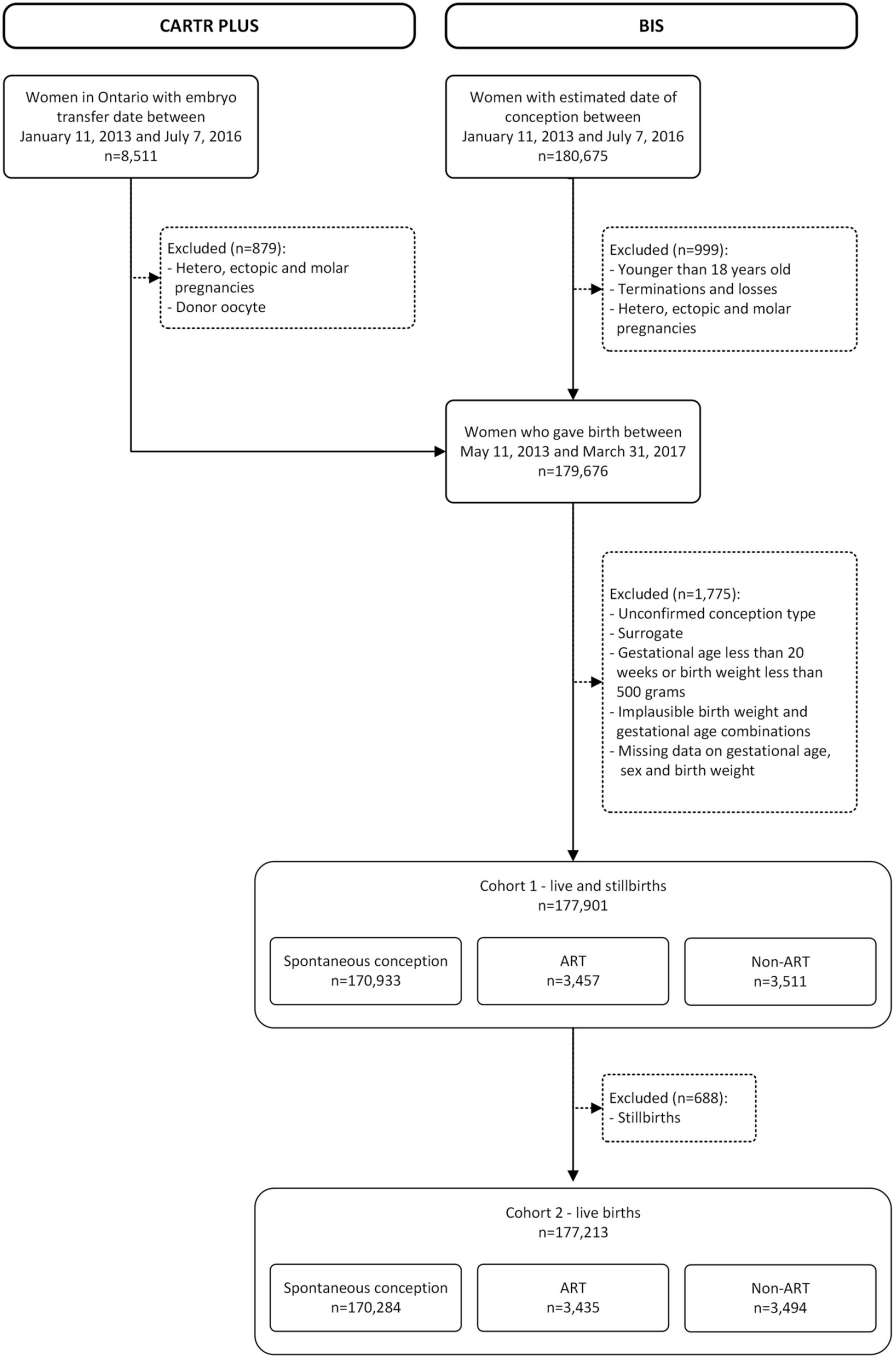
Flow diagram of study population. Abbreviations: ART – conception method referring to use of assisted reproductive technology; BIS – BORN Information System; CARTR Plus—Canadian Assisted Reproductive Technologies Register; Non-ART – conception method referring to use of ovulation induction, intra-uterine insemination and vaginal insemination

The study included 177,901 pregnant individuals (median gestation age (GA): 39 weeks [IQR 38.0–40.0]), 3,457 (1.9%) of whom conceived through ART (median GA: 38 weeks [IQR 37.0–40.0] and 3,511 (2.0%) with non-ART (median GA: 39 weeks [IQR 37.0–40.0]) (Fig. 1). In the unweighted study population (Table 1), pregnant individuals who underwent fertility treatments were more likely than those who conceived spontaneously to be nulliparous (ART 63.7%; non-ART 63.9%), and less likely to smoke (ART 1.1%; non-ART 2.1%), use illicit drugs (ART 0.2%; non-ART 0.5%) or consume alcohol during pregnancy (ART 1.1%; non-ART 1.3%). Women who conceived via ART were older in age (mean: 35.7 ± 4.6 years) than those who conceived spontaneously (mean: 31.1 ± 4.9 years) and were more likely to live in higher-income, higher-education neighbourhoods. Following propensity score weighting, the distribution of baseline characteristics was well balanced across groups (absolute standardized differences < 0.10), except for smoking during pregnancy comparing ART with spontaneously conceived pregnancies (standardized difference = 0.13). This covariate was included in the adjusted regression models to control for the imbalance between groups (doubly robust estimation) [38].

**Table 1:** Baseline characteristics of study population by method of conception, before and after average treatment effect (ATE) weighting (*n* = 177,901)

Characteristics	Unweighted cohort, n(%) of pregnancies						ATE-weighted cohort, % of pregnancies				
	**Total population**	**Spontaneous**	**ART**	**Non-ART**	**Standardized difference**^a^	**Standardized difference**^b^	**Spontaneous**	**ART**	**Non-ART**	**Standardized difference**^a,c^	**Standardized difference**^b,c^
	**(*n* = 177,901)**	**(*n* = 170,933)**	**(*n* = 3,457)**	**(*n* = 3,511)**							
**Maternal age (years)**											
Less than 30	61,636 (34.7)	60,677 (35.5)	282 (8.2)	677 (19.3)	**0.70**	**0.37**	34.7	31.0	34.2	0.08	0.01
30 to 34	70,679 (39.7)	68,030 (39.8)	1,132 (32.8)	1,517 (43.2)	**0.15**	0.07	39.7	41.3	39.7	0.03	0.00
35 to 39	38,018 (21.4)	35,574 (20.8)	1,412 (40.8)	1,032 (29.4)	**0.44**	**0.20**	21.4	23.1	21.9	0.04	0.01
40 to 42	6,109 (3.4)	5,472 (3.2)	385 (11.1)	252 (7.2)	**0.31**	**0.18**	3.4	3.8	3.5	0.02	0.00
43 and above	1,459 (0.8)	1,180 (0.7)	246 (7.1)	33 (0.9)	**0.34**	0.03	0.8	0.8	0.7	0.00	0.01
Mean age (SD)	31.27 (4.91)	31.14 (4.88)	35.65 (4.61)	33.20 (4.29)	**0.95**	**0.45**	31.25 (5.00)	32.37 (28.34)	31.78 (30.54)	0.05	0.02
**Neighbourhood education level**											
Quintile 1 (lowest)	21,984 (12.4)	21,437 (12.5)	191 (5.5)	356 (10.1)	**0.25**	0.08	13.1	11.4	12.3	0.05	0.02
Quintile 2	29,237 (16.4)	28,285 (16.6)	398 (11.5)	554 (15.8)	**0.15**	0.02	17.4	17.0	17.3	0.01	0.00
Quintile 3	37,629 (21.2)	36,133 (21.1)	717 (20.7)	779 (22.2)	0.01	0.03	22.4	22.2	22.2	0.00	0.00
Quintile 4	43,754 (24.6)	41,758 (24.4)	1,056 (30.6)	940 (26.8)	**0.14**	0.05	26.0	26.3	26.6	0.01	0.01
Quintile 5 (highest)	35,590 (20.0)	34,003 (19.9)	900 (26.0)	687 (19.6)	**0.15**	0.01	21.1	23.0	21.6	0.05	0.01
Missing	9,707 (5.5)	9,317 (5.5)	195 (5.6)	195 (5.6)	0.01	0.00	-	-	-	-	-
**Neighbourhood household income (median)**											
Quintile 1 (poorest)	31,892 (17.9)	31,118 (18.2)	348 (10.1)	426 (12.1)	**0.24**	**0.17**	19.1	16.0	16.8	0.08	0.06
Quintile 2	28,167 (15.8)	27,295 (16.0)	376 (10.9)	496 (14.1)	**0.15**	0.05	16.8	16.9	17.7	0.00	0.02
Quintile 3	31,603 (17.8)	30,347 (17.8)	605 (17.5)	651 (18.5)	0.01	0.02	18.9	18.7	19.4	0.01	0.01
Quintile 4	42,193 (23.7)	40,200 (23.5)	1,024 (29.6)	969 (27.6)	**0.14**	0.09	25.3	27.0	25.9	0.04	0.01
Quintile 5 (richest)	33,162 (18.6)	31,520 (18.4)	896 (25.9)	746 (21.3)	**0.18**	0.07	19.9	21.5	20.3	0.04	0.01
Missing	10,884 (6.1)	10,453 (6.1)	208 (6.0)	223 (6.4)	0.00	0.01	-	-	-	-	-
**Pre-pregnancy BMI**											
Non-obese	126,104 (70.9)	121,472 (71.1)	2,291 (66.3)	2,341 (66.7)	0.1	0.09	82.4	83.7	82.2	0.03	0.00
Obese	26,738 (15.0)	25,397 (14.9)	455 (13.2)	886 (25.2)	0.05	**0.26**	17.6	16.3	17.8	0.03	0.00
Missing	25,059 (14.1)	24,064 (14.1)	711 (20.6)	284 (8.1)	**0.17**	**0.19**	**-**	-	-	-	-
**Gravidity**											
1	59,114 (33.2)	56,161 (32.9)	1,359 (39.3)	1,594 (45.4)	**0.13**	**0.26**	33.9	35.6	35.0	0.04	0.02
≥ 2	115,519 (64.9)	111,641 (65.3)	1,996 (57.7)	1,882 (53.6)	**0.16**	**0.24**	66.1	64.4	65.1	0.04	0.02
Missing	3,268 (1.8)	3,131 (1.8)	102 (3.0)	35 (1.0)	0.07	0.07	-	-	-	-	-
**Parity**											
0	80,887 (45.5)	76,443 (44.7)	2,202 (63.7)	2,242 (63.9)	**0.39**	**0.39**	45.9	49.1	47.7	0.06	0.04
1	62,783 (35.3)	60,840 (35.6)	947 (27.4)	996 (28.4)	**0.18**	**0.16**	35.7	34.3	35.6	0.03	0.00
≥ 2	32,364 (18.2)	31,851 (18.6)	262 (7.6)	251 (7.2)	**0.33**	**0.35**	18.4	16.7	16.7	0.04	0.04
Missing	1,867 (1.1)	1,799 (1.1)	46 (1.3)	22 (0.6)	0.03	0.05	-	-	-	-	-
**Health conditions pre-pregnancy**											
No	141,841 (79.7)	137,224 (80.3)	2,312 (66.9)	2,305 (65.7)	**0.31**	**0.33**	77.5	76.7	77.7	0.02	0.01
Yes	36,060 (20.3)	33,709 (19.7)	1,145 (33.1)	1,206 (34.4)	**0.31**	**0.33**	22.5	23.4	22.3	0.02	0.01
Asthma^c^	6,446 (3.6)	6,170 (3.6)	90 (2.6)	186 (5.3)	0.06	0.08	4.2	3.9	3.7	0.02	0.03
Diabetes^c^	1,633 (0.9)	1,522 (0.9)	43 (1.2)	68 (1.9)	0.03	0.09	1.2	1.0	0.9	0.02	0.03
Chronic hypertension^c^	1,840 (1.0)	1,713 (1.0)	72 (2.1)	55 (1.6)	0.09	0.05	1.8	1.6	1.0	0.01	0.07
**Health complications during pregnancy**											
No	137,832 (77.5)	133,336 (78.0)	2,154 (62.3)	2,342 (66.7)	**0.35**	**0.25**	76.3	75.8	76.1	0.01	0.00
Yes	40,069 (22.5)	37,597 (22.0)	1,303 (37.7)	1,169 (33.3)	**0.35**	**0.25**	23.7	24.2	23.9	0.01	0.00
Gestational diabetes^d^	11,609 (6.5)	10,846 (6.3)	423 (12.2)	340 (9.7)	**0.20**	**0.12**	6.9	7.3	6.7	0.02	0.01
Hypertensive Disorders^d^	8,545 (4.8)	7,964 (4.7)	278 (8.0)	303 (8.6)	**0.14**	**0.16**	5.5	5.0	4.8	0.02	0.03
**Health behaviours during pregnancy**											
Smoking	9,830 (5.5)	9,719 (5.7)	38 (1.1)	73 (2.1)	**0.26**	**0.19**	6.8	3.9	5.5	**0.13**	0.05
Use of illicit drugs	2,242 (1.3)	2,219 (1.3)	6 (0.2)	17 (0.5)	**0.13**	0.09	2.5	1.8	1.7	0.05	0.05
Alcohol consumption	3,789 (2.1)	3,704 (2.2)	38 (1.1)	47 (1.3)	0.08	0.06	3.4	2.9	2.6	0.03	0.05
**Reason for IVF**											
Female structural only	331 (0.2)	-	331 (9.6)	-	-	-	-	-	-	-	-
Female hormonal only	415 (0.2)	-	415 (12.0)	-	-	-	-	-	-	-	-
Male only	682 (0.4)	-	682 (19.7)	-	-	-	-	-	-	-	-
Unexplained	409 (0.2)	-	409 (11.8)	-	-	-	-	-	-	-	-
Multiple or others	834 (0.5)	-	834 (24.1)	-	-	-	-	-	-	-	-
**Previous IVF cycle**											
0	786 (0.4)	-	786 (22.7)	-	-	-	-	-	-	-	-
≥ 1	1,885 (1.1)	-	1,885 (54.5)	-	-	-	-	-	-	-	-
**Infant Sex**											
Male	91,160 (51.2)	87,611 (51.3)	1,723 (49.8)	1,826 (52.0)	-	-	-	-	-	-	-
Female	86,741 (48.8)	83,322 (48.7)	1,734 (50.2)	1,685 (48.0)		-	-	-	-	-	-
**Median (IQR) gestational age (weeks)**	39.0 (38.0–40.0)	39.0 (38.0–40.0)	38.0 (37.0–39.0)	39.0 (37.0–40.0)	-	-	-	-	-	-	-
**Median (IQR) birthweight (grams)**	3,366 (3,030–3,695)	3,370 (3,040–3,700)	3,185 (2,680–3,570)	3,260 (2,835–3,610)	-	-	-	-	-	-	-

Assisted reproductive technology (ART) include in vitro fertilization (IVF), with or without intracytoplasmic sperm injection (ICSI)

Non-ART fertility treatments include ovulation induction, intra-uterine insemination and vaginal insemination

*Abbreviations: ART* Assisted reproductive technology, *ATE* Average treatment effect, *BMI* Body mass index, *IVF* In vitro fertilization, *SD* Standard deviation, *“- “* not applicable

^a^ Absolute standardized difference between ART and spontaneously conceived pregnancies. Numbers in bold indicate imbalance (> 0.10) between ART and spontaneously conceived pregnancies

^b^ Absolute standardized difference between non-ART and spontaneously conceived pregnancies. Numbers in bold indicate imbalance (> 0.10) between Non-ART and spontaneously conceived pregnancies

^c^ Data adjusted using average treatment effect (ATE) weights. Variables included maternal age, neighbourhood education level, neighbourhood household income, pre-pregnancy body mass index (BMI), gravidity, parity, pre-pregnancy health conditions (asthma, diabetes, chronic hypertension), health complications during pregnancy (gestational diabetes, hypertensive disorders), health behaviours during pregnancy (smoking, use of illicit drugs, alcohol consumption)

^d^ Sum of individual conditions does not equal total number of individuals with any condition, because categories were not mutually exclusive

### Pregnancy and birth outcomes

Crude cumulative incidences of adverse pregnancy and birth outcomes (except SGA) were highest in ART pregnancies (Table 2). Compared with spontaneously conceived pregnancies, ART pregnancies had significantly increased risks of stillbirth (aRR 2.26, 95% CI 2.04, 2.51), multiple birth (aRR 8.95, 95% CI 8.58, 9.34), cesarean delivery (aRR 1.44, 95% CI 1.42, 1.47), preterm birth (aRR 2.06, 95% CI 1.98, 2.14), very preterm birth (aRR 2.99, 95% CI 2.75, 3.25), and low Apgar score (aRR 1.28, 95% CI 1.16, 1.42). Within 28 days of birth, infants conceived via ART had a significantly increased risk of a diagnosis or procedure in the composite NAOI (aRR 1.61, 95% CI 1.55, 1.68). No association emerged between ART pregnancies and SGA in crude and adjusted analyses of live births only (RR 1.05, 95% CI 0.93, 1.17; aRR 0.98, 95% CI 0.95, 1.02).

**Table 2:** Association between fertility treatments and pregnancy and birth outcomes, Ontario, Canada (*n* = 177,901)

Outcome	Spontaneous		ART		Non-ART		ART		Non-ART	
	**No. of cases**	**Cumulative incidence (95% CI)**	**No. of cases**	**Cumulative incidence** **(95% CI)**	**No. of cases**	**Cumulative incidence** **(95% CI)**	**RR** **(95% CI)**	**aRR** **(95% CI)**^c^	**RR** **(95% CI)**	**aRR** **(95% CI)**^c^
**Cohort 1** *(live births and stillbirths)*	*n* = 170,933		*n* = 3,457		*n* = 3,511					
Stillbirth^a^	649	3.8 (3.51, 4.09)	22	6.36 (3.71, 9.01)	17	4.84 (2.55, 7.14)	1.68 (1.06, 2.49)	2.26 (2.04, 2.51)	1.28 (0.76, 1.99)	0.75 (0.64, 0.87)
Multiple birth	4,013	2.35 (2.27, 2.42)	761	22.01 (20.60, 23.42)	505	14.38 (13.20, 15.57)	9.38 (8.74, 10.05)	8.95 (8.58, 9.34)	6.13 (5.61, 6.67)	6.07 (5.84, 6.30)
Cesarean delivery	48,171	28.18 (27.96, 28.40)	1,686	48.77 (47.07, 50.47)	1,284	36.57 (34.95, 38.20)	1.73 (1.67, 1.79)	1.44 (1.42, 1.47)	1.3 (1.24, 1.36)	1.09 (1.07, 1.11)
Preterm	12,258	7.17 (7.05, 7.30)	714	20.65 (19.28, 22.03)	525	14.95 (13.75, 16.16)	2.88 (2.69, 3.08)	2.06 (1.98, 2.14)	2.09 (1.92, 2.26)	1.85 (1.79, 1.91)
Very preterm	1,923	1.13 (1.07, 1.18)	128	3.7 (3.06, 4.34)	105	2.99 (2.42, 3.57)	3.29 (2.75, 3.91)	2.99 (2.75, 3.25)	2.66 (2.18, 3.21)	1.89 (1.67, 2.13)
**Cohort 2** *(live births)*	*n* = 170,284		*n* = 3,435		*n* = 3,494					
SGA^b^	15,492	9.31 (9.17, 9.46)	261	9.74 (8.59, 10.88)	292	9.76 (8.67, 10.84)	1.05 (0.93, 1.17)	0.98 (0.95, 1.02)	1.05 (0.94, 1.17)	0.90 (0.87, 0.93)
Apgar 5 (< 7)	2,995	1.78 (1.72, 1.85)	100	2.97 (2.39, 3.56)	102	2.94 (2.37, 3.51)	1.67 (1.36, 2.02)	1.28 (1.16, 1.42)	1.65 (1.35, 1.99)	1.62 (1.45, 1.81)
Composite NAOI	12,466	7.32 (7.19, 7.45)	501	14.59 (13.38, 15.79)	411	11.76 (10.67, 12.85)	1.99 (1.83, 2.16)	1.61 (1.55, 1.68)	1.61 (1.46, 1.76)	1.29 (1.25, 1.34)

Assisted reproductive technology (ART) include in vitro fertilization (IVF), with or without intracytoplasmic sperm injection (ICSI)

Non-ART fertility treatments include ovulation induction, intra-uterine insemination and vaginal insemination

*Abbreviations: 95% CI* 95% Confidence interval, *ART* Assisted reproductive technology, *NAOI* Neonatal adverse outcome indicator, *No.* Number, *RR* Risk ratio, *aRR* Adjusted risk ratio, *SGA* Small for gestational age

^a^ Cumulative incidence per 1,000 women

^b^ Singletons only

^c^ Data adjusted using average treatment effect (ATE) weights. Variables included maternal age, neighbourhood education level, neighbourhood household income, pre-pregnancy body mass index (BMI), gravidity, parity, pre-pregnancy health conditions (asthma, diabetes, chronic hypertension), health complications during pregnancy (gestational diabetes, hypertensive disorders), health behaviours during pregnancy (smoking, use of illicit drugs, alcohol consumption)

The risks of adverse pregnancy and birth outcomes were significantly higher for non-ART pregnancies, compared with spontaneously conceived pregnancies—multiple birth (aRR 6.07, 95% CI 5.84, 6.30), cesarean delivery (aRR 1.09, 95% CI 1.07, 1.11), preterm birth (aRR 1.85, 95% CI 1.79, 1.91), very preterm birth (aRR 1.89, 95% CI 1.67, 2.13), low Apgar score (aRR 1.62, 95% CI 1.45, 1.81), and composite NAOI (aRR 1.29, 95% CI 1.25, 1.34). By contrast, such pregnancies were associated with significantly reduced risks of stillbirth (aRR 0.75, 95% CI 0.64, 0.87) and SGA (aRR 0.90, 95% CI 0.87, 0.93).

### Infant health outcomes

The cumulative incidence of NICU admission was higher among infants conceived via ART (5.59%) and via non-ART (4.15%), compared with spontaneously conceived infants (2.21%) (Table 3). After adjustment, the likelihood of NICU admission for more than 24 h was significantly increased for ART (aRR 1.98, 95% CI 1.84, 2.13) and non-ART infants (aRR 1.59, 95% CI 1.51, 1.67). Additionally, being conceived through ART (aRR 1.60, 95% CI 1.54, 1.65) and non-ART (aRR 1.42, 95% CI 1.39, 1.45) was significantly associated with longer LOS during birth admission. The incidence rate of all-cause urgent and inpatient health services use was higher among non-ART conceived infants (2.93 per 1,000 person-days) compared with spontaneously conceived counterparts (2.75 per 1,000 person days), and a small but significantly high rate of all-cause urgent and inpatient health services use during the first year of life was apparent after adjustment (aIRR 1.10, 95% CI 1.08, 1.13). Similarly, an increased rate was observed among ART infants after adjustment (aIRR 1.06, 95% CI 1.04, 1.09).

**Table 3:** Association between fertility treatments and pediatric health outcomes, Ontario, Canada (*n* = 177,213)

Outcome	Spontaneous (*n* = 170,284)		ART (*n* = 3,435)		Non-ART (*n* = 3,494)		ART		Non-ART	
	**No. of events**	**Incidence rate (95% CI) per 1,000 person-days**	**No. of events**	**Incidence rate (95% CI) per 1,000 person-days**	**No. of events**	**Incidence rate (95% CI) per 1,000 person-days**	**IRR** **(95% CI)**	**aIRR**^b^ **(95% CI)**	**IRR** **(95% CI)**	**aIRR**^b^ **(95% CI)**
**Non-specific infant health services use**										
Admission to NICU (> 24 h)^a^	3,763	2.21 (2.14, 2.28)	192	5.59 (4.81, 6.37)	145	4.15 (3.48, 4.82)	2.53 (2.19, 2.90)	1.98 (1.84, 2.13)	1.88 (1.59, 2.20)	1.59 (1.51, 1.67)
3 or more days of hospital stay (birth admission)^a^	20,930	12.29 (12.13, 12.45)	939	27.34 (25.82, 28.86)	771	22.07 (20.66, 23.47)	2.22 (2.10, 2.35)	1.60 (1.54, 1.65)	1.80 (1.68, 1.91)	1.42 (1.39, 1.45)
Urgent and inpatient health services use (1st year)	171,036	2.75 (2.74, 2.77)	3,285	2.62 (2.53, 2.71)	3,729	2.93 (2.83, 3.02)	0.95 (0.91, 1.00)	1.06 (1.04, 1.09)	1.06 (1.01, 1.11)	1.10 (1.08, 1.13)
**Disease-specific infant health services use**										
Upper respiratory tract infections	26,945	3.52 (3.48, 3.56)	399	3.38 (3.07, 3.73)	491	3.40 (3.11, 3.71)	0.96 (0.92, 1.01)	1.00 (0.98, 1.02)	0.97 (0.92, 1.01)	0.97 (0.96, 0.99)
Lower respiratory tract infections	17,727	4.19 (4.13, 4.25)	262	3.97 (3.51, 4.48)	351	4.13 (3.72, 4.58)	0.95 (0.87, 1.03)	0.98 (0.96, 1.01)	0.99 (0.92, 1.06)	1.05 (1.00, 1.10)
Gastrointestinal infections	8,517	3.29 (3.22, 3.36)	160	3.27 (2.80, 3.82)	193	3.3 (2.87, 3.81)	0.99 (0.93, 1.06)	1.06 (1.03, 1.10)	1.00 (0.95, 1.07)	1.06 (1.04, 1.09)
Otitis media	8,687	3.27 (3.20, 3.33)	98	3.12 (2.56, 3.81)	176	3.09 (2.67, 3.58)	0.96 (0.88, 1.04)	0.92 (0.91, 0.94)	0.95 (0.89, 1.01)	0.97 (0.94, 1.01)
All infections	61,876	4.69 (4.65, 4.72)	919	4.22 (3.96, 4.49)	1,211	4.6 (4.36, 4.86)	0.9 (0.85, 0.95)	0.96 (0.93, 0.98)	0.98 (0.94, 1.03)	1.05 (1.03, 1.07)
**Infant health outcomes**										
Pediatric complex chronic conditions (PCCC)^a,c^	609	0.36 (0.33, 0.39)	23	0.67 (0.39, 0.95)	17	0.49 (0.25, 0.72)	1.87 (1.20, 2.76)	2.38 (2.03, 2.78)	1.36 (0.81, 2.13)	0.78 (0.67, 0.92)
Infant death (1st year)^a,d^	156	0.09 (0.08, 0.11)	6	0.17 (0.03, 0.32)	6	0.17 (0.03, 0.31)	1.91 (0.75, 3.94)	1.58 (0.72, 3.47)	1.87 (0.74, 3.87)	1.21 (0.94, 1.56)

Assisted reproductive technology (ART) include in vitro fertilization (IVF), with or without intracytoplasmic sperm injection (ICSI)

Non-ART fertility treatments include ovulation induction, intra-uterine insemination and vaginal insemination

*Abbreviations: ART* Assisted reproductive technology, *95% CI* 95% Confidence interval, *IRR* Incidence rate ratio, *aIRR* Adjusted incidence rate ratio, *NICU* Neonatal intensive care unit

^a^ Cumulative incidence; point estimates are risk ratios generated using log binomial regression model

^b^ Data adjusted using average treatment effect (ATE) weights. Variables included maternal age, neighbourhood education level, neighbourhood household income, pre-pregnancy body mass index (BMI), gravidity, parity, pre-pregnancy health conditions (asthma, diabetes, chronic hypertension), health complications during pregnancy (gestational diabetes, hypertensive disorders), health behaviours during pregnancy (smoking, use of illicit drugs, alcohol consumption)

^c^ Number of events comprises total unweighted number of children diagnosed as having two or more of pediatric complex chronic conditions (PCCC). We derived the PCCC indicator based on the following categories: neurologic and neuromuscular; cardiovascular; respiratory; renal and urologic; gastrointestinal; hematologic or immunologic; metabolic; other congenital or genetic defect; malignancy; and technology assistance

^d^ Analysis based on number of deaths during first year of life, excluding those that occurred on date of birth

In crude analyses, we did not observe associations between use of fertility treatments and disease-related health services use. However, after adjustment, risks of gastrointestinal infections were significantly increased for ART (aIRR 1.06, 95% CI 1.03, 1.10) and non-ART (aIRR 1.06, 95% CI 1.04, 1.09). We observed small risk reductions for upper respiratory tract infections among non-ART pregnancies (aIRR 0.97, 95% CI 0.96, 0.99), and for otitis media among ART pregnancies (aIRR 0.92 95% CI 0.91, 0.94).

ART pregnancies were significantly associated with complex chronic conditions by age one (aRR 2.38, 95% CI 2.03, 2.78), while the risk for non-ART pregnancies was significantly reduced (aRR 0.78, 95% CI 0.67, 0.92). No association with infant mortality emerged for pregnancies via ART (aRR 1.58, 95% CI 0.72, 3.47) or non-ART (aRR 1.21, 95% CI 0.94, 1.56).

### Sensitivity analysis

Restricting analyses to singletons showed significantly increased risks generally persisted for ART pregnancies, except for low Apgar score with reduced risk (aRR 0.71, 95% CI 0.64, 0.78) (see Additional file 6). Significantly increased risks also persisted for non-specific infant health services use outcomes when the analysis was further restricted to term singletons (see Additional file 7). Overall, the magnitude of risks was lower among this subgroup of singletons and term singletons than in the full study cohort.

## Discussion

In this study, fertility treatments were associated with significantly increased risks for most adverse pregnancy and birth outcomes (multiple birth, cesarean delivery, preterm, very preterm, low Apgar score and composite NAOI), compared with spontaneously conceived pregnancies. As well, infants conceived via ART and non-ART fertility treatments had higher rates of urgent and inpatient health services use during their first year. These results persisted, but were slightly attenuated, when the analyses were restricted to singletons (pregnancy and birth outcomes) and term singletons (infant health outcomes).

Consistent with our findings, several studies with ART (IVF and IVF-ICSI) and/or stand-alone use of non-ART fertility treatments as distinct exposure group(s) found significantly increased risk of preterm and very preterm birth [42–49], cesarean delivery [42], low Apgar score [13, 47], and composite neonatal morbidity [13]. Higher risks were observed among ART pregnancies than non-ART pregnancies [43, 44]. A study of 57,624 pregnancies in Quebec found a 76% increased risk of preterm birth among IVF pregnancies versus 47% among pregnancies achieved through ovulation induction [43]. Our analysis of singletons yielded results consistent with Klemetti et al. who reported that increased odds of preterm and very preterm birth after ovulation induction were attenuated for singletons [47].

In contrast to earlier studies [45, 46], we observed no association between ART and SGA, and a small reduction in the risk of SGA for non-ART, compared with spontaneously conceived infants. This could reflect different gestational age assessments in the groups—last menstrual period for spontaneous conceptions versus (1) exact embryo transfer date for ART, and (2) validated last menstrual period through follicle monitoring for non-ART. However, we cannot rule out the possibility that residual confounding by factors associated with both fertility treatments and SGA (e.g., maternal smoking) played a role, despite the use of propensity score methods for adjustment.

Numerous studies have examined health services use by infants conceived via fertility treatments [13, 22, 44, 45, 48, 50]. A randomized US clinical trial of 460 newborn twins found an increased risk of NICU admission among those conceived via IVF/ICSI, compared with spontaneously conceived twins (aRR 1.27, 95% CI 1.003, 1.60). A large register-based Belgian study also reported a higher risk of NICU admission among IVF singletons [44]. In Sweden, Källén et al. observed a 73% increase in rates of hospitalization among IVF births, compared with those conceived spontaneously; the elevated risk fell to 44% when the analysis was limited to term births [22].

Similar results reported in this study, a recent Norwegian study with 84,102 singleton children (spontaneously conceived *n* = 74,867 and conceived via ART *n* = 1,901) found no increased risk of upper and lower respiratory infections during the first 18 months, which can be partly explain by breastfeeding during the early months of life and its protective role against infections [51]. On the contrary, we observed a reduction in risk of upper respiratory infections and otitis media among infants born after non-ART and ART procedures, respectively. The “precious baby” effect [52] could be one potential reason for why an inverse association was seen between fertility treatments and pediatric infections. It has been shown that one’s health-seeking behaviour is associated with socio-economic status and patients who have undergone fertility treatments have higher education and income levels and are more likely to know how to navigate the health care system. Infertile or subfertile parents were highly conscious of their children’s health and may have sought more frequent medical care compared to fertile parents. Although we adjusted for socio-economic status in our models to account for health-seeking behaviour, the possibility of residual confounding remains.

Lastly, we observed an increased risk of stillbirth and PCCC among infants born after ART and a reduced risk of these outcomes among non-ART infants. Direct comparison of our results with other studies was difficult because many of them restricted their data to singleton pregnancies and used variable outcome definitions (i.e., stillbirth from 20 weeks’ gestation vs. 22 weeks; stillbirth vs. perinatal mortality) [45, 46, 48, 53, 54]. Our results may have been affected by both plurality and prematurity; however, we could not conduct subgroup analyses stratified by these factors due to the small numbers of these outcomes in the ART and non-ART groups.

The main strength of this study is the use of a population-based birth registry data, linked to an ART registry and other health administrative datasets. Validation studies of the BIS and CARTR Plus databases have shown high accuracy of key maternal and birth data [30, 31, 33, 55]; therefore, we expect minimal misclassification of exposure and outcomes. Use of province-wide population-based databases minimized potential selection bias and ensure high external validity.

Our results have several limitations. Subgroup analyses may be underpowered for uncommon outcomes such as stillbirth and infant mortality. Information bias due to misclassification of specific diseases is possible. Limiting analyses of health care use to hospitals and emergency departments (versus venues such as physicians’ offices) biased our results toward more serious outcomes. We lacked information on children who lost eligibility for provincial health care coverage owing to death or out-of-province migration. These factors reduced the sensitivity of our outcome measurement and biased our estimates toward the null value, given that we hypothesized that it would be non-differential by exposure. Although our data sources had comprehensive sociodemographic, clinical and health care information, we cannot rule out potential residual confounding, such as the underlying cause of infertility.

## Conclusion

We found that pregnancies conceived via fertility treatments were at increased risk of adverse pregnancy, birth, and infant health outcomes. The magnitude of risk was greater for pregnancies conceived using ART rather than non-ART fertility treatments. It may be warranted for clinicians to counsel patients about the implications of fertility treatments. In addition, healthcare providers and policymakers need to consider the consequences of increased health care use by infants exposed to fertility treatments.

## Supplementary Information

**Additional file 1: Supplementary Figure 1.** Timeline of eligible conceptions and corresponding births.

**Additional file 2: Supplementary Table 1.** Components of Neonatal Adverse Outcome Indicator (NAOI) and corresponding BORN Information System, ICD-10-CA and CCI codes.

**Additional file 3: Supplementary Table 2.** Categories of disease-specific health services utilization and corresponding ICD-10-CA codes.

**Additional file 4: Supplementary Table 3.** Categories of Pediatric Complex Chronic Conditions (PCCC) and corresponding ICD-10-CA codes.

**Additional file 5: Supplementary Table 4.** RECORD checklist.

**Additional file 6: Supplementary Table 5.** Association between fertility treatments and pregnancy and birth outcomes (singletons only), Ontario, Canada (*n*=172,622).

**Additional file 7: Supplementary Table 6.** Association between fertility treatments and infant health outcomes (term singletons only), Ontario, Canada (*n*=161,887).

## Data Availability

The dataset from this study is held securely by BORN Ontario. Although legal data sharing agreements between BORN Ontario and data providers (e.g., health care organizations and government) prohibit BORN Ontario from making the dataset publicly available, the analytic code may be available on request. Inquiries regarding the data used in this study or the analytic code can be directed to dfell@cheo.on.ca.

## Abbreviations

aIRR: Adjusted incidence rate ratios
aRR: Adjusted risk ratios
ART: Assisted reproductive technology
ATE: Average treatment effect
BORN: Better Outcomes Registry & Network
BIS: BORN Information System
BMI: Body mass index
CARTR Plus: Canadian Assisted Reproductive Technologies Register Plus
CI: Confidence intervals
CIHI: Canadian Institute for Health Information
DAD: Discharge Abstract Database
FCS: Fully conditional specification
GA: Gestational age
GBM: Generalized boosted model
ICD-10-CA: Canadian version of International Classification of Diseases, 10th Revision
IRR: Unadjusted incidence rate ratios
IVF: In vitro fertilization
NACRS: National Ambulatory Care Reporting System
NAOI: Neonatal adverse outcome indicator
NICU: Neonatal intensive care unit
PCCC: Pediatric complex chronic conditions
PHIPA: Personal Health Information Protection Act
RECORD: REporting of studies Conducted using Observational Routinely-collected Data
RR: Unadjusted risk ratios
SGA: Small for gestational age
TWANG: Toolkit for Weighting and Analysis of Non-equivalent Groups
