# Supplementary figures and images for: Perinatal and pediatric outcomes associated with the use of fertility treatment: a population-based retrospective cohort study in Ontario, Canada
Source: BMC Pregnancy Childbirth. 2023 Feb 20;23:121. doi: 10.1186/s12884-023-05446-3 (PMC9940338; doi:10.1186/s12884-023-05446-3)

**Supplementary figure 1. Timeline of eligible conceptions and corresponding births**

**
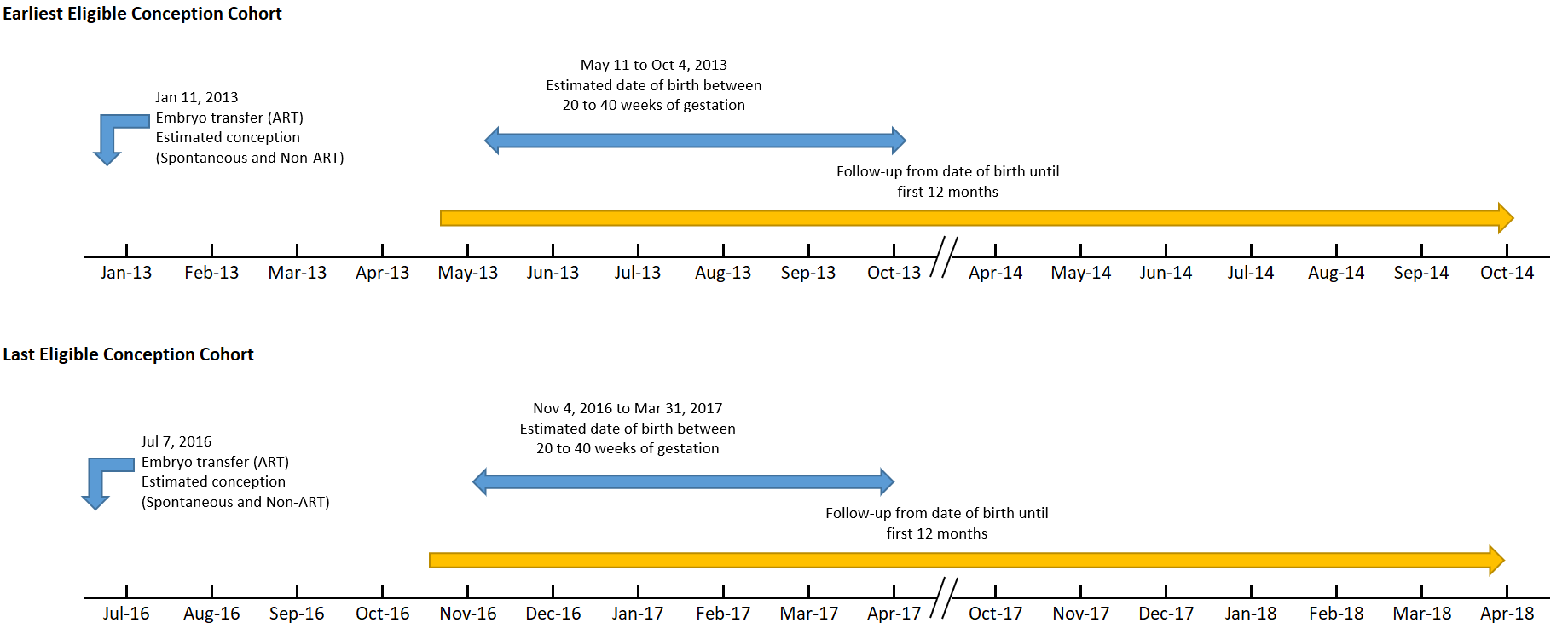
**

Supplement: Supplementary file 1 — Additional file 1: Supplementary Figure 1. Timeline of eligible conceptions and corresponding births. [file 12884_2023_5446_MOESM1_ESM.docx]
