# Supplementary material for: Perinatal and pediatric outcomes associated with the use of fertility treatment: a population-based retrospective cohort study in Ontario, Canada
Source: BMC Pregnancy Childbirth. 2023 Feb 20;23:121. doi: 10.1186/s12884-023-05446-3 (PMC9940338; doi:10.1186/s12884-023-05446-3)
Supplement: Supplementary file 2 — Additional file 2: Supplementary Table 1. Components of Neonatal Adverse Outcome Indicator (NAOI) and corresponding BORN Information System, ICD-10-CA and CCI codes. [file 12884_2023_5446_MOESM2_ESM.docx]

# **Supplementary table 1. Components of Neonatal Adverse Outcome Indicator (NAOI) and corresponding BORN Information System, ICD-10-CA and CCI codes** (1)

| **Neonatal Adverse Outcome Indicator (NAOI)** | **BORN Information System variable** | **DAD diagnosis and procedure codes (ICD-10-CA & CCI)** |
| --- | --- | --- |
| **Diagnosis** | | |
| Gestational age <32 weeks | N0014 | - |
| Birthweight <1500 grams | N0012 | - |
| Neonatal mortality |  | Discharge disposition: 07, 08 |
| Respiratory Distress Syndrome |  | ICD-10_CA: P220 |
| Seizure |  | ICD-10_CA: P90, R56 |
| Intraventricular hemorrhage (grades 2, 3, or 4) |  | ICD-10_CA: P521, P522 |
| Cerebral infarction |  | ICD-10_CA: I63 |
| Periventricular leukomalacia |  | ICD-10_CA: P912 |
| Birth trauma |  | ICD-10_CA: P100 TO P103, P130, P132, P133, P140, P141 |
| Hypoxic ischemic encephalopathy |  | ICD-10_CA: P915, P9181, P916 |
| Necrotising enterocolitis |  | ICD-10_CA: P77 |
| Bronchopulmonary dysplasia |  | ICD-10_CA: P271 |
| Sepsis/septicaemia |  | ICD-10_CA: P36 |
| Pneumonia |  | ICD-10_CA: P23 |
| Other respiratory: primary atelectasis, respiratory failure |  | ICD-10_CA: P280, P285 |
| **Procedure** | | |
| Any body cavity surgical procedure  (substring) |  | CCI: 1AA52, 1AA87, 1AC87, 1AE87, 1AF87, 1AG87, 1AJ87, 1AK87, 1AN52, 1AN59, 1AN87, 1AP59, 1AP72, 1AP87, 1AW59, 1AW72, 1AW87, 1AX87, 1BA72, 1BA80, 1BA87, 1BB72, 1BB80, 1BB87, 1BD72, 1BD80, 1BD87, 1BF80, 1BG72, 1BG80, 1BG87, 1BK59, 1BM72, 1BM80, 1BM87, 1BN72, 1BN80, 1BN87, 1BP72, 1BP80, 1BP87, 1BQ72, 1BQ80, 1BQ87, 1BS72, 1BS80, 1BS87, 1BT72, 1BT80, |
| **Neonatal Adverse Outcome Indicator (NAOI)** | **BORN Information System variable** | **DAD diagnosis and procedure codes (ICD-10-CA & CCI)** |
|  |  | 1BT87, 1GA87, 1GA89, 1GB87, 1GB89, 1GD89, 1GE80, 1GE87, 1GE89, 1GE91, 1GH84, 1GJ86, 1GJ87, 1GK87, 1GK89, 1GM80, 1GM86, 1GM87, 1GN92, 1GR87, 1GR89, 1GR91, 1GT78, 1GT87, 1GT89, 1GT91, 1GV87, 1GV89, 1GW87, 1GX80, 1GX86, 1GX87, 1GY70, 1GY72, 1GY86, 1HJ76, 1HJ82, 1HN87, 1HP76, 1HP78, 1HP80, 1HP82, 1HP83, 1HP87, 1HR80, 1HR84, 1HR87, 1HS80 (excluding 1HS80G), 1HS90, 1HT80 (excluding 1HT80G), 1HT89, 1HT90, 1HU80 (excluding 1HU80G), 1HU90, 1HV80 (excluding 1HV80G), 1HV90, 1HW78, 1HW79, 1HX80, 1HX87, 1HX80, 1HZ87, 1IA76, 1IA80, 1IA87, 1IB76, 1IB79, 1IB80, 1IB82, 1IB87, 1IC76, 1IC80, 1IC82, 1IC87, 1ID76, 1ID80, 1ID82, 1ID86, 1ID87, 1IF83, 1IJ76, 1IJ80, 1IM76, 1IM80, 1IM82, 1IM83, 1IM87, 1IN83, 1IN84, 1IN87, 1JE57 (excluding 1JE57G), 1JE76, 1JE80, 1JE87, 1JJ76, 1JJ80, 1JK76, 1JK80, 1JK87, 1JW51 (excluding 1JW51G), 1JW57, 1JW76, 1LA84, 1LC84, 1LD84, 1NA72, 1NA74, 1NA76, 1NA77, 1NA80, 1NA84, 1NA86, 1NA87, 1NA88, 1NA89, 1NA90, 1NA91, 1NA92, 1NE80, 1NF76, 1NF78, 1NF80, 1NF82, 1NF84, 1NF86, 1NF87 (excluding 1NF87B), 1NF89, 1NF90, 1NF91, 1NF92, 1NK76, 1NK77, 1NK80, 1NK82, 1NK84, 1NK87 (excluding 1NK87B), 1NM74, 1NM76, 1NM77, 1NM80, 1NM82, 1NM87 (excluding 1NM87B), 1NM89, 1NM91, 1NP72, 1NP73, 1NP86, 1NQ74 (excluding 1NQ74B), 1NQ80, 1NQ84, 1NQ86, 1NQ87 (excluding 1NQ87B), 1NQ89, 1NQ90, 1NT80, 1NT84, 1NT86, 1NT87, 1NV89, 1OA87, 1OB87, 1OB89, 1OD76, 1OD89, 1OE76, 1OE80, 1OE89, 1OJ76 (excluding 1OJ76B), 1OJ87, 1OJ89, 1OK87, 1OK89, 1OK91, 1OT72, 1OT87, 1OT91, 1PB87, 1PB89, 1PC80, 1PC87 (excluding 1PC87D), 1PC89, 1PC91, 1PE57 (excluding 1PE57BD), 1PE80 (excluding 1PE80D), 1PE82, 1PE87 (excluding 1PE87D), 1PE89 |
| **Neonatal Adverse Outcome Indicator (NAOI)** | **BORN Information System variable** | **DAD diagnosis and procedure codes (ICD-10-CA & CCI)** |
|  |  | (excluding 1PE89D), 1PG76, 1PG77, 1PG80 (excluding 1PG80D), 1PG86, 1PG89, 1PL74 (excluding 1PL74CD), 1PL80, 1PM79, 1PM86, 1PM87 (excluding 1PM87B), 1PM89, 1PM90, 1PM91, 1PM92, 1QE53, 1QE80, 1QE82, 1QE84, 1QE87, 1QE89, 1QG89, 1QM74, 1QM80, 1QM87, 1QM89, 1QM91, 1QN82, 1QT87, 1QT91, 1RB74, 1RB80, 1RB83, 1RB87, 1RB89,1RD89, 1RF51, 1RF72, 1RF74, 1RF80, 1RF87, 1RF89, 1RM87 (excluding 1RM87B), 1RM89, 1RM91, 1RN87, 1RN89, 1RS74, 1RS80, 1RS86, 1RS87, 1RS89, 1RW87, 1RW88, 1RW91, 1RW92, 1SA74, 1SA75, 1SA80, 1SA89, 1SC74, 1SC75, 1SC80, 1SC87, 1SC89, 1SE53, 1SE89 (excluding 1SE89D), 1SF80, 1SF87, 1SF89, 1SG80, 1SG87, 1SH87, 1SM74, 1SM80, 1SM87, 1SN87, 1SN93, 1SQ53, 1SQ74, 1SQ80, 1SQ87, 1SQ91, 1SQ93, 1SW74, 1SY80, 1SY84, 1SY87, 1SZ87, 1VA53, 1VA74, 1VA75, 1VA80, 1VA87, 1VA93, 1VC74, 1VC80, 1VC87, 1VC91, 1VC93, 1VE80, 1VG53, 1VG55, 1VG72, 1VG73, 1VG74, 1VG75, 1VG80, 1VG87, 1VG93, 1VK80, 1VK87, 1VK89, 1VL80, 1VL87, 1VM80, 1VM87, 1VN80, 1VN87, 1VP74, 1VP80, 1VP87, 1VP89, 1VQ74, 1VQ79, 1VQ80, 1VQ82, 1VQ87, 1VQ91, 1VQ93, 1VS72, 1VS80, 1VX87 |
| Any intravenous fluids |  | CCI: 1LZ35CAE6, 1LZ35HAC1, 1LZ35HAC5, 1LZ35HAC6, 1LZ35HAC7, 1LZ35HAE6, 1LZ35HAT7, 1LZ35HAT9, 1LZ35HAZ9, 1LZ35HHC1, 1LZ35HHC5, 1LZ35HHC6, 1LZ35HHC7, 1LZ35HHE0, 1LZ35HHE6, 1LZ35HHT7, 1LZ35HHT9, 1LZ35HHZ9, 1LZ35HRC5, 1LZ35HRC6, 1LZ35HRC7, 1LZ35HRT9, 1LZ35HRZ9 |
| Central venous or arterial catheter |  | CCI: 1KV53HACH, 1KV53HAFT, 1KV53LAFT, 2IM28GP, 2LZ28GQPL, 2LZ28GRPL, 2LZ28JAPL, 1KX53HACH, 1KX53HAFT, 1KX53 LAFT, 2LZ28GQPL, 2LZ28GRPL |
| **Neonatal Adverse Outcome Indicator (NAOI)** | **BORN Information System variable** | **DAD diagnosis and procedure codes (ICD-10-CA & CCI)** |
| Pneumothorax requiring intercostal catheter |  | CCI: 1GV52DA, 1GV52DATS, 1GV52HA, 1GV52HAHE, 1GV52HATK, 1GV52LA, 1GV52LATS, 1GV52LAXXE, 1GV54JATS, 1GV55JATS |
| Resuscitation |  | CCI: 1HZ30JN, 1HZ30JY, 1GZ30CJ, 1GZ30CJNB, 1GZ30JH |
| Transfusion of blood or blood products |  | CCI: 1LZ19HHU1A, 1LZ19HHU1J, 1LZ19HHU2A, 1LZ19HHU2J, 1LZ19HHU3J, 1LZ19HHU4J, 1LZ19HHU5J, 1LZ19HHU6A, 1LZ19HHU6J, 1LZ19HHU9A, 1LZ19HHU9J, 1LZ19HMU1, 1LZ19HMU2, 1LZ19HMU9, 1LZ35HAC5 |
| Ventilatory support (mechanical ventilation and/or CPAP) |  | CCI: 1GZ31CAEP, 1GZ31CAND, 1GZ31CAPK, 1GZ31CBND, 1GZ31CRND, 1GZ31GPND, 1GZ31JAGX, 1GZ31JAMD, 1GZ31JANC, 1GZ31JAPK |
|  |  |  |

**Reference:**

1. Wanigaratne S, Cole DC, Bassil K, Hyman I, Moineddin R, Shakya Y, Urquia ML. Severe Neonatal Morbidity Among Births to Refugee Women. Matern Child Health J. 2016;20(10):2189-98.
