# Supplementary material for: Perinatal and pediatric outcomes associated with the use of fertility treatment: a population-based retrospective cohort study in Ontario, Canada
Source: BMC Pregnancy Childbirth. 2023 Feb 20;23:121. doi: 10.1186/s12884-023-05446-3 (PMC9940338; doi:10.1186/s12884-023-05446-3)
Supplement: Supplementary file 3 — Additional file 3: Supplementary Table 2. Categories of disease-specific health services utilization and corresponding ICD-10-CA codes. [file 12884_2023_5446_MOESM3_ESM.docx]

# **Supplementary table 2. Categories of disease-specific health services utilization and corresponding ICD-10-CA codes** (1)

| **Infection-related pediatric health outcomes** | **ICD-10-CA diagnostic codes resulting from any hospital admission or emergency department record from birth to 12 months of age** |
| --- | --- |
| Upper respiratory tract infections | A36.0, A36.1, A36.2, A36.8, A36.9, J01-J06, J35.0, J36, J37.0 |
| Lower respiratory tract infections | A37, A42.0, A48.1, A70, J09-J18, J20-J22, J85, J86 |
| Gastrointestinal infections | A00, A01, A02.0, A02.2-A02.9, A03-A09, A42.1 |
| Otitis media | H65 to H67 |
| Composite of infections | All infection codes listed above |

# **Reference:**

1. Walsh LK, Donelle J, Dodds L, Hawken S, Wilson K, Benchimol EI, Chakraborty P, Guttmann A, Kwong JC, MacDonald NE, Ortiz JR, Sprague AE, Top KA, Walker MC, Wen SW, Fell DB. Health outcomes of young children born to mothers who received 2009 pandemic H1N1 influenza vaccination during pregnancy: retrospective cohort study. BMJ. 2019;366:l4151.
