# Supplementary material for: Perinatal and pediatric outcomes associated with the use of fertility treatment: a population-based retrospective cohort study in Ontario, Canada
Source: BMC Pregnancy Childbirth. 2023 Feb 20;23:121. doi: 10.1186/s12884-023-05446-3 (PMC9940338; doi:10.1186/s12884-023-05446-3)
Supplement: Supplementary file 4 — Additional file 4: Supplementary Table 3. Categories of Pediatric Complex Chronic Conditions (PCCC) and corresponding ICD-10-CA codes. [file 12884_2023_5446_MOESM4_ESM.docx]

# **Supplementary table 3. Categories of Pediatric Complex Chronic Conditions (PCCC) and corresponding ICD-10-CA codes** (1,2)

| **Complex Chronic Conditions** | **ICD-10-CA diagnostic codes resulting from any hospital admission or emergency department record from birth to 12 months of age** |
| --- | --- |
| Neurologic / Neuromuscular | E750, E751, E752, E754, F71, F72, F73, F842, G111, G112, G114, G118, G119, G120, G121, G122, G128, G129, G3101, G3109, G318, G3189, G3289, G71, G72, G80, G901, G938, G939, G94, Q00, Q01, Q02, Q03, Q04, Q05, Q06, Q07, G911, G319, G253, G9519, G9589, G909, G40311, G40301, G40211, G40219, G40411, G40419, G40804, G40111, G40119, G40911, G40919, G371, G372, G378, G8190, G8290, G8250, G8251, G8252, G8253, G8254, G835, G839, G931, G935, I6330, I6350, G10, G20, G210, G2111, G2119, G218, G230, G231, G232, G238, G2402, G248, G253, G254, G255, G2581, G2582, G2583, G2589, G259, G803, R403, G9782, Z982, Z4541, Z4542, G310, G328, G951, G958, G4031, G4030, G4021, G4041, G4080, G4011, G4091, G82, I633, I635, G211, G240, G258, R4020, G978, G113, G318, Q851 |
| Cardiovascular | I270, I271, I272, I2781, I2789, I279, I340, I348, I360, I368, I370, I378, I42, I43, I44, I45, I47, I48, I490, I491, I493, I494, I495, I498, I499, I509, I515, I517, I5181, I63139, I63239, Q20, Q212, Q213, Q214, Q218, Q22, Q23, Q24, Q251, Q252, Q253, Q254, Q255, Q256, Q257, Q26, R001, Q282, Q283, Q289, Z951, Z941, Z950, Z952, Z95810, Z95811, Z95812, Z95818, Z953, Z45010, Z45018, Z4502, Z4509, Z959, I278, I518, I631, I632, T86200, T86201, T86202, T86300, T86301, T86302, Q219, Q258, Q259, Z943 |
| Respiratory | E84, G4753, I2782, I43, Q30, Q31, Q32, Q33, Q34, P280, Z902, J84112, J9500, J9501, J9502, J9503, J9504, J9509, J9620, Z430, Z930, Z942, Z990, J95850, Z9911, Z9912, T86819, I278, J841, J9503, J96, J95, T86800, T86801, T86802 |
| Urorenal | N18, Q60, Q61, Q62, Q63, Q64, Z905, Z906, G834, N312, N319, Z940, Z9350, Z9351, Z9352, Z9359, Z936, Z9115, Z992, Z435, Z436, Z446, Z4901, Z4902, Z4931, Z4932, T8610, T8611, T8612, Z49, Z490 |
| Gastrointestinal | K50, K51, K73, K74, K754, K760, K761, K762, K763, K768, Q390, Q391, Q392, Q393, Q394, Q41, Q42, Q43, Q44, Q45, I820, K551, K562, K593, Z980, Z903, Z9049, K9420, K9422, K9423, K9429, Z944, Z9482, Z9483, Z931, Z932, Z933, Z934, Z431, Z432, Z433, Z434, Z4651, Z4659, T8640, T8641, T8642, T86890, T86891, T86899, T86850, T86851, T86859, Z904, K765, T86810, T86811, T86812 |
| Hematology and immunodeficiency | B20, D55, D56, D57, D58, D60, D61, D71, D720, D80, D81, D82, D83, D84, D85, D87, D88, D86, M303, M359, B21, B22, B23, B24, D700, D704, D66, D682, D6941, D6942, D761, D762, D763, D869, M300, M310, M311, M3130, M314, M316, M3210, M3390, M340, M341, M349, D694, M321, M339, D89, D694 |
| Metabolic | D841, E700, E702, E703, E704, E705, E708, E710, E711, E712, E713, E714, E715, E720, E721, E722, E723, E724, E728, E729, E740, E741, E742, E743, E744, E748, E749, E75, E760, E761, E762, E763, E770, E771, E780, E781, E782, E783, E784, E785, E786, E787, E788, E789, E791, E798, E804, E805, E806, E807, E830,E831, E833, E834, E88, H498, E85, E009, E230, E232, E222, E233, E237, E240, E242, E243, E248, E249, E2681, E250, E258, E259, Z4681, Z794, Z9641, E70, E71, E78, E268 |
| **Complex Chronic Conditions** | **ICD-10-CA diagnostic codes resulting from any hospital admission or emergency department record from birth to 12 months of age** |
| Other Congenital or Genetic Defect | E343, K449, M410, M412, M4130, M418, M419, M4330, M965, Q722, Q750, Q752, Q759, Q760, Q761, Q762, Q764, Q765, Q766, Q767, Q77, Q780, Q781, Q782, Q783, Q784, Q788, Q789, Q790, Q791, Q792, Q793, Q794, Q799, Q795, Q8740, Q8781, Q8789, Q897, Q899, Q909, Q913, Q914, Q917, Q928, Q93, Q950, Q969, Q97, Q98, Q992, Q998, Q999, Q898, Q81 |
| Malignancy | C00, C01, C02, C03, C04, C05, C06, C07, C08, C09, C10, C11, C12, C13, C14, C15, C16, C17, C18, C19, C20, C21, C22, C23, C24, C25, C26, C30, C31, C32, C33, C34, C35, C36, C37, C38, C39, C40, C41, C43, C44, C45, C46, C47, C48, C49, C50, C51, C52, C53, C54, C55, C56, C57, C58, C60, C61, C62, C63, C64, C65, C66, C67, C68, C69, C70, C71, C72, C73, C74, C75, C76, C77, C78, C79, C80, C81, C82, C83, C84, C85, C86, C87, C88, C89, C90, C91, C92, C93, C94, C95, C96, C97, D00, D01, D02, D03, D04, D05, D06, D07, D08, D09, D10, D11, D12, D13, D14, D15, D16, D17, D18, D19, D20, D21, D22, D23, D24, D25, D26,D27, D28, D29, D30, D31, D32, D33, D34, D35, D36, D37, D38, D39, D40, D41, D42, D43, D44, D45, D46, D47, D48 |
| Technology Assistance | K9140, K9141, K9142, K9149, J9500, J9501, J9502, J9503, J9508, J9509, Z459, Z930, Z931, Z932, Z933, Z935, Z936, Z430, Z431, Z432, Z433, Z435, Z436, Z491, Z492, Z992, Z465, Z950, Z96, T823, T824 |

**References:**

1. Feudtner C, Feinstein JA, Zhong W, Hall M, Dai D. Pediatric complex chronic conditions classification system version 2: updated for ICD-10 and complex medical technology dependence and transplantation. BMC Pediatr. 2014;14:199.
2. Cohen E, Berry JG, Camacho X, Anderson G, Wodchis W, Guttmann A. Patterns and costs of health care use of children with medical complexity. Pediatrics. 2012;130(6):e1463-70.
