# Supplementary material for: Perinatal and pediatric outcomes associated with the use of fertility treatment: a population-based retrospective cohort study in Ontario, Canada
Source: BMC Pregnancy Childbirth. 2023 Feb 20;23:121. doi: 10.1186/s12884-023-05446-3 (PMC9940338; doi:10.1186/s12884-023-05446-3)
Supplement: Supplementary file 6 — Additional file 6: Supplementary Table 5. Association between fertility treatments and pregnancy and birth outcomes (singletons only), Ontario, Canada (n=172,622). [file 12884_2023_5446_MOESM6_ESM.docx]

# **Supplementary table 5. Association between fertility treatments and pregnancy and birth outcomes (singletons only), Ontario, Canada (n=172,622)**

| **Outcome** | **Spontaneous** | | | | **ART** | | | **Non-ART** | | |  | **ART** | | | **Non-ART** | |
| --- | --- | --- | --- | --- | --- | --- | --- | --- | --- | --- | --- | --- | --- | --- | --- | --- |
|  | **No. of cases** | | | **Cumulative incidence (95% CI)** | **No. of cases** | **Cumulative incidence**  **(95% CI)** | | **No. of cases** | **Cumulative incidence (95% CI)** | |  | **RR**  **(95% CI)** | **aRR**  **(95% CI)** ^a^ | | **RR**  **(95% CI)** | **aRR**  **(95% CI)** ^a^ |
| **Cohort 1**  *(live births and stillbirths)* | | | n=166,920 | | | | n=2,696 | | | n=3,006 | | | |  | | |
| Cesarean delivery | 45,860 | | | 27.47  (27.26, 27.69) | 1,146 | 42.51  (40.60, 44.41) | | 989 | 32.9  (31.19, 34.61) | |  | 1.55  (1.48, 1.62) | 1.23  (1.21, 1.26) | | 1.20  (1.14, 1.26) | 1.02  (1.00, 1.04) |
| Preterm | 10,034 | | | 6.01  (5.89, 6.13) | 278 | 10.31  (9.14, 11.48) | | 242 | 8.05  (7.06, 9.04) | |  | 1.72  (1.53, 1.92) | 1.21  (1.15, 1.27) | | 1.34  (1.18, 1.51) | 1.12  (1.06, 1.19) |
| Very preterm | 1,583 | | | 0.95  (0.90, 1.00) | 57 | 2.11  (1.56, 2.67) | | 57 | 1.9  (1.40, 2.39) | |  | 2.23  (1.70, 2.87) | 1.83  (1.66, 2.02) | | 2.00  (1.52, 2.57) | 1.42  (1.15, 1.75) |
| **Cohort 2**  *(live births)* | | n=166,327 | | | | | n=2,680 | | | n=2,992 | | | |  | | |
| Apgar 5 (<7) | 2,804 | | | 1.71  (1.64, 1.77) | 51 | 1.94  (1.40, 2.48) | | 78 | 2.62  (2.04, 3.21) | |  | 1.14  (0.85, 1.48) | 0.71  (0.64, 0.78) | | 1.54  (1.22, 1.90) | 1.65  (1.45, 1.88) |
| Composite NAOI | 11,408 | | | 6.86  (6.73, 6.98) | 284 | 10.6  (9.41, 11.79) | | 268 | 8.96  (7.91, 10.00) | |  | 1.55  (1.38, 1.72) | 1.22  (1.18, 1.26) | | 1.31  (1.16, 1.46) | 1.10  (1.05, 1.15) |

Abbreviations: 95% CI – 95% Confidence interval; ART – Assisted reproductive technology; NAOI - neonatal adverse outcome indicator; No. – Number; RR – Risk ratio; aRR – Adjusted risk ratio.

Assisted reproductive technology (ART) include in vitro fertilization (IVF), with or without intracytoplasmic sperm injection (ICSI).

Non-ART fertility treatments include ovulation induction, intra-uterine insemination and vaginal insemination.

^a^ Data adjusted using average treatment effect (ATE) weights. Variables included maternal age, neighbourhood education level, neighbourhood household income, pre-pregnancy body mass index (BMI), gravidity, parity, pre-pregnancy health conditions (asthma, diabetes, chronic hypertension), health complications during pregnancy (gestational diabetes, hypertensive disorders), adverse health behaviours during pregnancy (smoking, use of illicit drugs, alcohol consumption).
