# Supplementary material for: Perinatal and pediatric outcomes associated with the use of fertility treatment: a population-based retrospective cohort study in Ontario, Canada
Source: BMC Pregnancy Childbirth. 2023 Feb 20;23:121. doi: 10.1186/s12884-023-05446-3 (PMC9940338; doi:10.1186/s12884-023-05446-3)
Supplement: Supplementary file 7 — Additional file 7: Supplementary Table 6. Association between fertility treatments and infant health outcomes (term singletons only), Ontario, Canada (n=161,887). [file 12884_2023_5446_MOESM7_ESM.docx]

# **Supplementary table 6. Association between fertility treatments and infant health outcomes (term singletons only), Ontario, Canada (n=161,887)**

| **Outcome** | **Spontaneous**  **(n=156,713)** | | **ART**  **(n=2,412)** | | **Non-ART**  **(n=2,762)** | |  | **ART** | | **Non-ART** | |
| --- | --- | --- | --- | --- | --- | --- | --- | --- | --- | --- | --- |
|  | **No. of events** | **Incidence Rate (95% CI) per 1,000 person-days** | **No. of events** | **Incidence Rate (95% CI) per 1,000 person-days** | **No. of events** | **Incidence Rate (95% CI) per 1,000 person-days** |  | **IRR**  **(95% CI)** | **aIRR**  **(95% CI)** | **IRR**  **(95% CI)^b^** | **aIRR**  **(95% CI)^b^** |
| **Non-specific infant health services use** | | | | | | | | | | | |
| Admission to NICU (>24 hours)^a^ | 1,727 | 1.10  (1.05, 1.15) | 41 | 1.70  (1.18, 2.22) | 42 | 1.52  (1.06, 1.98) |  | 1.54  (1.13, 2.10) | 1.29  (1.18, 1.41) | 1.13  (1.02, 1.87) | 1.39  (1.20, 1.61) |
| 3 or more days of hospital stay (birth admission)^a^ | 13,390 | 8.54  (8.41, 8.68) | 318 | 13.18  (11.83, 14.53) | 340 | 12.31  (11.08, 13.54) |  | 1.54  (1.39, 1.71) | 1.08  (1.04, 1.12) | 1.44  (1.30, 1.59) | 1.08  (1.05, 1.11) |
| Urgent and inpatient health  services use (1^st^ year) | 151,178 | 2.64  (2.62, 2.66) | 2,187 | 2.49  (2.34, 2.64) | 2,856 | 2.83  (2.69, 2.99) |  | 0.94  (0.89, 1.00) | 1.04  (1.01, 1.07) | 1.07  (1.02, 1.13) | 1.14  (1.12, 1.16) |

Abbreviations: 95% CI – 95% Confidence interval; ART – Assisted reproductive technology; IRR – Incidence rate ratio; aIRR – Adjusted incidence rate ratio; NICU – Neonatal intensive care unit; No. – Number.

Assisted reproductive technology (ART) include in vitro fertilization (IVF), with or without intracytoplasmic sperm injection (ICSI).

Non-ART fertility treatments include ovulation induction, intra-uterine insemination and vaginal insemination.

^a^ Cumulative incidence; point estimates are risk ratios generated using log binomial regression model.

^b^ Data adjusted using average treatment effect (ATE) weights. Variables included maternal age, neighbourhood education level, neighbourhood household income, pre-pregnancy body mass index (BMI), gravidity, parity, pre-pregnancy health conditions (asthma, diabetes, chronic hypertension), health complications during pregnancy (gestational diabetes, hypertensive disorders), adverse health behaviours during pregnancy (smoking, use of illicit drugs, alcohol consumption).
